# Pre- and Post-Endurance Training Mitigates the Rat Pilocarpine-Induced Status Epilepticus and Epileptogenesis-Associated Deleterious Consequences

**DOI:** 10.3390/ijms232113188

**Published:** 2022-10-29

**Authors:** Michaela Shishmanova-Doseva, Katerina Georgieva, Yordanka Uzunova, Lyubka Ioanidu, Milena Atanasova, Zlatina Nenchovska, Jana Tchekalarova

**Affiliations:** 1 Department of Pharmacology, Toxicology and Pharmacotherapy, Faculty of Pharmacy, Medical University of Plovdiv, 15A Vassil Aprilov Blvd, 4002 Plovdiv, Bulgaria; 2 Department of Physiology, Faculty of Medicine, Medical University of Plovdiv, 15A Vassil Aprilov Blvd, 4002 Plovdiv, Bulgaria; 3 Department of Bioorganic Chemistry, Faculty of Pharmacy, Medical University of Plovdiv, 15A Vassil Aprilov Blvd, 4002 Plovdiv, Bulgaria; 4 Research Institute, Medical University of Plovdiv, 15A Vassil Aprilov Blvd, 4002 Plovdiv, Bulgaria; 5 Department of Biology, Medical University of Pleven, 5800 Pleven, Bulgaria; 6 Institute of Neurobiology, Bulgarian Academy of Sciences (BAS), 1113 Sofia, Bulgaria

**Keywords:** aerobic training, epilepsy, cognitive and mood comorbidities, oxidative stress, neuroinflammation

## Abstract

Epilepsy is a brain disorder characterized by recurrent epileptic seizures and neurobiological, physiological, mood, and cognitive consequences. In the last decade, the beneficial effects of regular physical exercise have been investigated in patients with neurodegenerative diseases such as epilepsy. However, data on its beneficial effects and underlying mechanisms are still insufficient. The objective of the current study was to investigate the effects of endurance training, applied before and after pilocarpine (Pilo) administration, on status epilepticus (SE) severity, and its relation to epileptogenesis deleterious consequences during the chronic epileptic phase. Long-term aerobic training, applied four weeks before SE and eight weeks after SE, elevated the threshold to induce SE and reduced spontaneous motor seizures. The protective effect of this alternative approach on seizure susceptibility resulted in improved memory responses, and alleviated comorbid depression in epileptic rats. The exercised epileptic rats had improved markers of oxidative stress by decreasing lipid peroxidation and increasing the levels of glutathione and activity of superoxide dismutase in the rat hippocampus. Aerobic training managed to ameliorate the neuroinflammation by decreasing the levels of TNF-α and IL-1β in the hippocampus. Our results suggest that regular physical training predisposes the subjects to crucial plastic changes, leading to increased resistance to SE and the development of epileptogenesis.

## 1. Introduction

Epilepsy is a heterogeneous brain disorder affecting 1 to 3% of the population worldwide. The main symptom of epilepsy is the onset of spontaneous seizures resulting from an impaired excitatory/inhibitory balance [1]. Temporal lobe epilepsy (TLE) is the most frequent type of epilepsy, and the most refractory type to drug treatment [2]. Often this disorder is associated with memory decline, emotional disturbance, and social consequences, which can negatively affect the quality of life of epileptic patients [3].

The pharmacological approach is the most common therapeutic tool to control epilepsy and seizure-related deleterious consequences on brain function. However, the latter can also cause many side effects, amongst which cognitive and behavioral impairments are the most frequently observed [4]. Although the participation of people with epilepsy in different sports activities has been widely debated over the last years, the beneficial effects of exercise on physiological and psychological outcomes are well established nowadays [5]. A growing number of clinical and experimental data has revealed that regular physical activity can reduce the interictal epileptiform activity and seizure frequency [6], decrease seizure susceptibility and threshold, as well as delay the development of amygdala kindling [7,8,9]. Moreover, additional benefits of aerobic training for epileptic patients can be similar to those of healthy individuals, such as increased work and maximal aerobic capacity, reduced heart rate, improved cardiovascular function, ameliorated cognate function, weight reduction, increased bone health, etc. [10,11,12,13]. Furthermore, regular physical exercises can improve mental health by relieving the symptoms of depression and anxiety, which are, on the other hand, the most common comorbidities in epilepsy [14]. Due to all of these observations, physical exercise can now be recommended as a complementary non-pharmacological approach to treating epilepsy. According to every patient’s clinical condition, the most suitable training program must be prepared [9].

Since clinical studies cannot provide sufficient information about the precise mechanisms underlying seizure susceptibility and epileptogenesis, animal models for epilepsy are a reliable approach for such mechanistic studies [1]. Pilocarpine (Pilo) is one of the most widely used convulsant agents that induce status epilepticus (SE), followed by neurochemical alterations and structural and functional changes similar to those observed in epileptic patients [15]. The hippocampus is the main target region where repeated seizures cause changes in neuronal excitability and cell death. Its dysfunction is a primary pathophysiologic mechanism of epileptic seizure [16]. Although the precise mechanism of the beneficial effects of aerobic training has not yet been fully elucidated, recent studies revealed improved neurogenesis, increased neurotrophins, inhibited apoptosis, and modulation of several neurotransmitter systems, such as glutamatergic, serotoninergic, dopaminergic, and noradrenergic [13,17,18,19]. Moreover, abnormalities in these mechanisms participate not only in the propagation and progression of epilepsy, but also in the occurrence of its comorbidities [14]. Recent studies have shown that oxidative stress and inflammatory cytokines are also involved in the pathogenesis of epilepsy and in mood and cognitive disturbances. However, data on the effect of aerobic training on these mechanisms is still scarce [20,21].

Previous reports used a protocol with exercise treatment to mitigate SE, mainly before or after its onset [22]. In the present study, we applied endurance training, for the first time, both before and after the Pilo-induced SE. This alternative approach was used to alleviate epileptogenesis and to suppress the development of a chronic state, or its consequences on the frequency of spontaneous motor seizures (SMS) and behavioral impairments in a rat model of post-SE-induced TLE. In addition, the levels of the pro-inflammatory cytokines tumor necrosis factor-α (TNF-α) and interleukin-1β (IL-1β), as well as oxidative stress markers, in the hippocampus were measured.

## 2. Results

### 2.1. The Endurance Training, Applied before and after SE, Mitigated the Pilocarpine-Induced Status Epilepticus in the Rat

After a single dose of 320 mg/kg pilocarpine, all sedentary rats developed sustained motor seizures at stages IV and V, respectively, during the first hour after SE onset. Only seven rats (44%) from the exercised group reached stage IV during the first hour. The other animals from the exercised group reached only stages I and II and presented with mouth and facial movements and head nodding during the first hour, so they received a booster dose of 160 mg/kg pilocarpine to develop IV and V stage seizures. During SE, 20% of the sedentary group died, while no mortality was detected in the exercised group.

### 2.2. The Endurance Training, Applied before and after SE, Alleviated SMS in the Pilo-Induced Post-SE Rat Model of TLE

**Figure 1:**
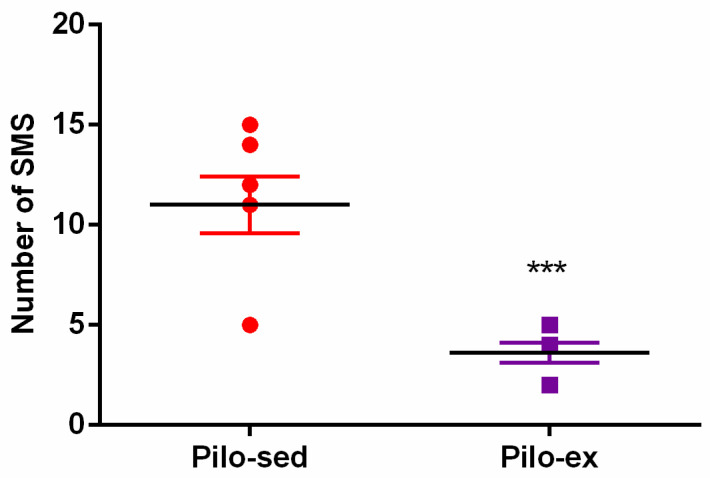
The effect of endurance training, applied before and after SE, on the total number of spontaneous motor seizures (SMS) in a pilocarpine-induced post-status epilepticus model of temporal lobe epilepsy. *** *p* < 0.05 in comparison with the Pilo-sed group.

Video recording was used 24 h a day for seven days in sixteen rats (n = 8 from the Pilo-sed and Pilo-ex groups) two months after SE. The SMS were detected in all rats from both the Pilo-sed and Pilo-ex group, respectively. During the chronic phase of epilepsy endurance training, applied before and after the Pi-lo-induced SE, the frequency of seizures was reduced. The SMS were significantly decreased in the Pilo-ex group in comparison with the Pilo-sed group (Student *t*-test: *p* ≤ 0.05) (Figure 1).

### 2.3. Behavioral Tests

#### 2.3.1. The Endurance Training, Applied before and after SE, Improved the Formation of Short- and Long-Term Memory Traces in the Step-Down Test in the Pilo-Induced Post-SE Rat Model of TLE

**Figure 2:**
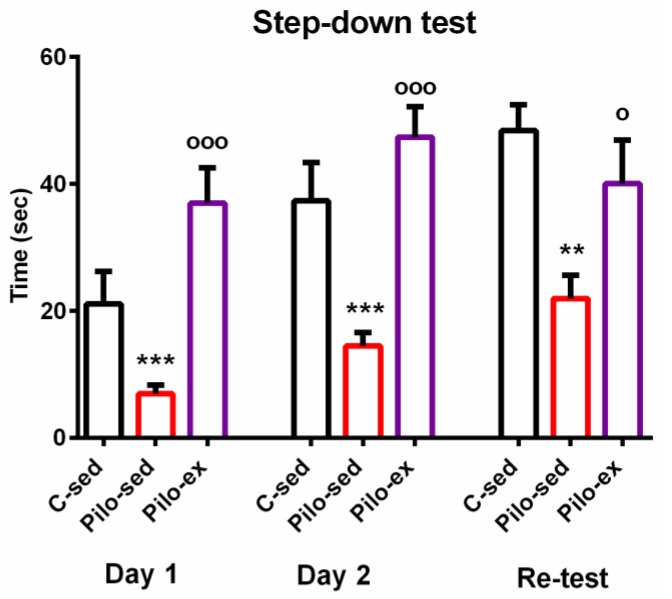
The effect of endurance training, applied before and after SE, on the latency reaction during the first, second, and re-test days in the step-down test in the pilocarpine-induced post-status epilepticus model of temporal lobe epilepsy. ** *p* = 0.003, *** *p* < 0.05 in comparison with the C-sed group; ^ooo^ *p* < 0.001, ^o^ *p* = 0.046 in comparison with the Pilo-sed group.

ANOVA showed a main effect of exercise [F(2, 23) = 10.866, *p* = 0.001] on the learning session during the first day of the passive avoidance test. The post hoc test indicated that both groups C-sed and Pilo-ex had a longer latency reaction than the Pilo-sed animals (*p* = 0.043 and *p* < 0.001, respectively) (Figure 2).

The main effect of exercise [F(2, 23) = 13.595, *p* = 0.001] was observed during the test for short-term memory traces. The post hoc test showed that the Pilo-sed group spent a shorter time on the platform compared to the Pilo-ex group and the C-sed group (*p* < 0.001 and *p* = 0.001, respectively).

A main effect of exercise [F(2, 23) = 7.288, *p* = 0.004] was revealed during the test for long-term memory traces. ANOVA showed that the Pilo-sed animals had a significantly shorter latency reaction in comparison with the C-sed rats (*p* = 0.003) and the Pilo-ex group (*p* = 0.046). There were no significant differences in the measured indices be-tween the Pilo-ex and C-sed animals.

#### 2.3.2. The Endurance Training, Applied before and after SE, Corrected the Impairment of Associative Memory in the Object Recognition Test in the Pilo-Induced Post-SE Rat Model of TLE

**Figure 3:**
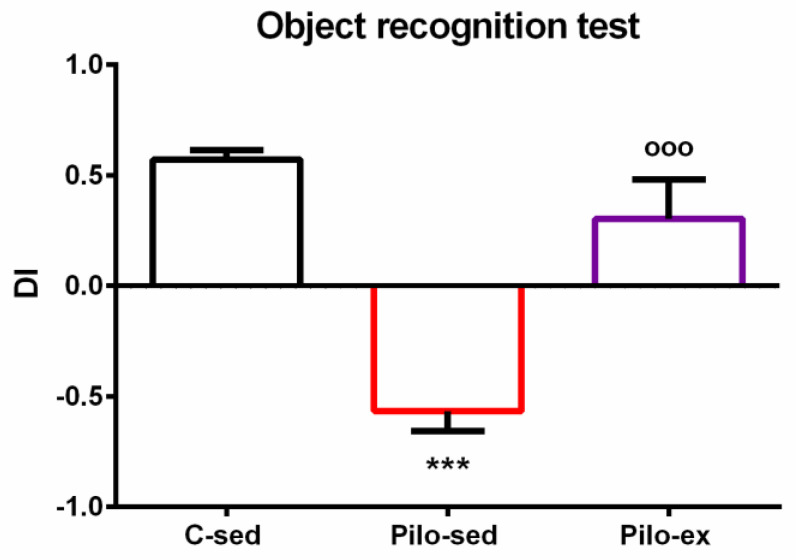
The effect of endurance training, applied before and after SE, on the discrimination index in the pilocarpine-induced post-SE model of TLE. *** *p* < 0.001 in comparison with the C-sed group; ^ooo^ *p* < 0.001, in comparison with the Pilo-sed group.

ANOVA revealed a main effect of exercise [F(2, 23) = 25.843, *p* < 0.001] on the dis-crimination index. The post hoc test showed that the Pilo-sed animals had impaired object recognition memory compared to both the C-sed and Pilo-ex groups (*p* < 0.001) (Figure 3).

#### 2.3.3. The Endurance Training, Applied before and after SE, Improved Spatial Memory in the Eight-Radial Arm Maze Test in the Pilo-Induced Post-SE Rat Model of TLE

**Figure 4:**
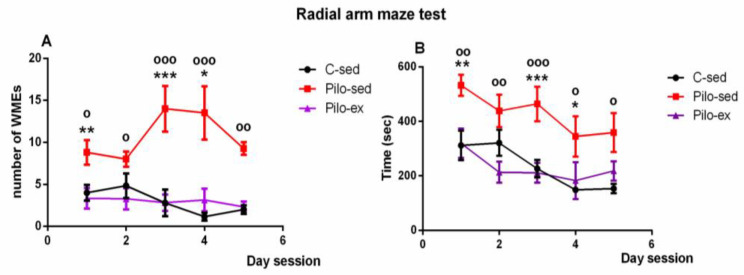
Effect of endurance training, applied before and after SE, on (**A**) the number of working memory errors; * *p* = 0.047, ** *p* = 0.007, *** *p* = 0.001 in comparison with the C-sed group; ^o^ *p* < 0.05, ^oo^ *p* = 0.002, ^ooo^ *p* < 0.001 in comparison with the Pilo-sed group, and (**B**) on the time (s) necessary to fulfill the task in the radial arm maze test in a pilocarpine-induced model of temporal lobe epilepsy. * *p* < 0.05, ** *p* < 0.01, *** *p* < 0.001 in comparison with the C-sed group; ^o^ *p* < 0.05, ^oo^ *p* < 0.01, ^ooo^ *p* < 0.001 in comparison with the Pilo-sed group.

A significant effect of time [F(4, 119) = 4.823, *p* = 0.002] and Exercise [F(2, 119) = 26,296, *p* < 0.001] on the number of working memory errors (WMEs) was detected. The Pilo-ex group had WMEs similar to control group number (*p* > 0.05), while the Pilo-sed group had significantly more WMEs in comparison with the C-sed animals during the 1st, 3rd, and 4th sessions (*p* = 0.007, *p* = 0.001, and *p* = 0.047, respectively) and in comparison with the Pilo-ex animals (1st, 2nd, 3rd, 4th, and 5th sessions) (*p* = 0.015, *p* = 0.035, *p* < 0.001, *p* < 0.001, and *p* = 0.002, respectively) (Figure 4A).

Analysis of variance demonstrated a significant main effect of time [F(4, 119) = 4.823, *p* = 0.002) and exercise [F(1, 119) = 26.293, *p* < 0.001] for the time necessary to fulfill the task. The Pilo-ex group had time similar to that of the control animals (*p* > 0.05). The Pilo-sed group needed more time to fulfill the task in comparison with the C-sed rats (1st, 3rd, and 4th session) (*p* = 0.007, *p* < 0.001, and *p* = 0.047, respectively) and in comparison with the Pilo-ex group (1st, 2nd, 3rd, 4th, and 5th session) (*p* = 0.01, *p* = 0.006, *p* < 0.001, *p* = 0.038, and *p* = 0.01) (Figure 4B).

#### 2.3.4. The Endurance Training, Applied before and after SE, Failed to Correct the Pilocarpine-Induced Impairment on Anxiety Response in the Open Field Test in the Pilo-Induced Post-SE Rat Model of TLE

**Figure 5:**
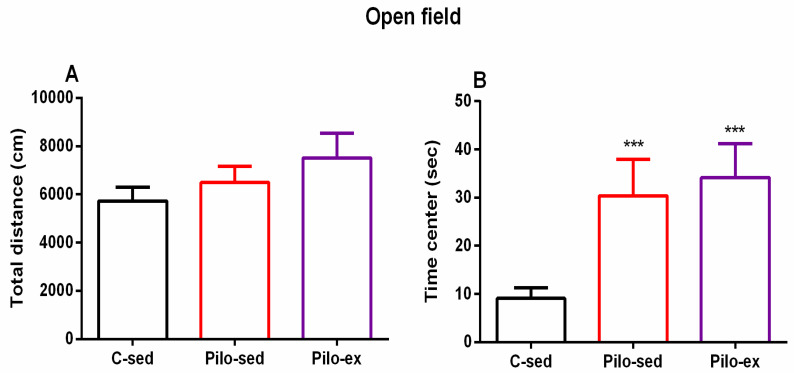
Effect of endurance training, applied before and after SE, on (**A**) total distance and (**B**) on the time (s) in the center in the open field test. *** *p* < 0.001 in comparison with the C-sed group.

No significant difference between the three groups was observed concerning the locomotor activity in the novel field (*p* > 0.05) (Figure 5A). ANOVA revealed a main effect of exercise [F(2, 23) = 11.16, *p* < 0.001] on the time spent in the aversive central zone of the open field. The post hoc test showed that both groups Pilo-sed and Pilo-ex had lower anxiety levels compared to the C-sed group (*p* < 0.001) (Figure 5B).

#### 2.3.5. Endurance Training, Applied before and after SE, Alleviated the Anxiety-Like Behavior in the Elevated Plus Maze Test (EPM) in the Pilo-Induced Post-SE Rat Model of TLE

**Figure 6:**
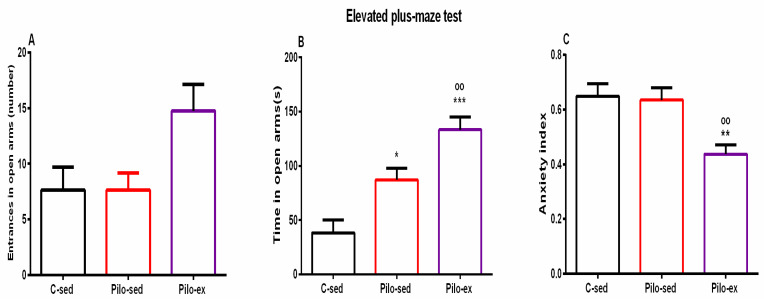
Effect of endurance training, applied before and after SE, on (**A**) number of entrances in open arms, (**B**) time (s) in open arms, * *p* = 0.017, *** *p* = 0.001, respectively, in comparison with the C-sed group, ^oo^ *p* = 0.024, in comparison with the Pilo-sed group, (**C**) ** *p* = 0.005 in comparison with the C-sed group, ^oo^ *p* = 0.009 in comparison with the Pilo-sed group.

ANOVA revealed a significant main effect of exercise [F(2, 23) = 4.031, *p* = 0.033] on the number of entries in the open arms of EPM. There was a tendency for a higher number of entries in the open arms of EPM of the Pilo-ex group compared to the C-sed and the Pilo-sed animals (*p* = 0.057 and *p* = 0.059, respectively) (Figure 6A).

Analysis of variance demonstrated a significant main effect of exercise [F(2, 23) = 17.311, *p* < 0.01] on time spent in the open arms of the EPM. Both groups treated with Pilo had decreased levels of anxiety, and spent a longer time in the aversive area in comparison with the C-sed animals (*p* = 0.017 and *p* = 0.001, respectively) (Figure 6B). Moreover, endurance training enhanced this impulsive-like behavior (*p* = 0.024 compared to the Pilo-sed group) (Figure 6B).

A significant main effect was demonstrated for exercise [F(2, 23) = 7.804, *p* = 0.003] on the anxiety index. The post hoc test demonstrated that the Pilo-ex rats had the lowest anxiety index compared to the C-sed (*p* = 0.005) and the Pilo-sed groups (*p* = 0.009) (Figure 6C).

#### 2.3.6. Endurance Training, Applied before and after SE, Alleviated the Depressive-Like Behavior in the Pilo-Induced Post-SE Rat Model of TLE

**Figure 7:**
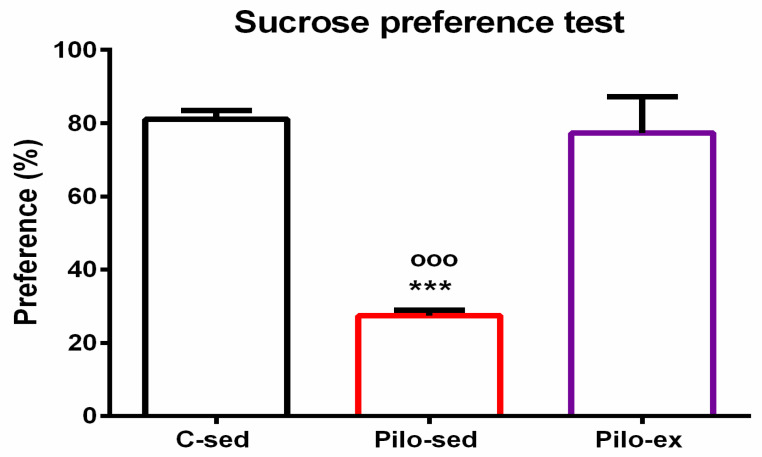
Effect of endurance training, applied before and after SE, on preference to sucrose solution in the sucrose preference test, *** *p* < 0.001 and ^ooo^ *p* < 0.001 in comparison with the C-sed group and Pilo-sed group, respectively.

For the sucrose preference test, analysis of variance showed a significant main effect of exercise [F(2, 23) = 25.638, *p* < 0.001]. The post hoc test showed that the Pilo-sed animals had anhedonia in comparison with the C-sed and Pilo-ex groups (*p* < 0.001, respectively) (Figure 7).

### 2.4. Endurance Training, Applied before and after SE, Mitigated the Oxidative Stress in the Hippocampus in the Pilo-Induced Post-SE Rat Model of TLE

**Figure 8:**
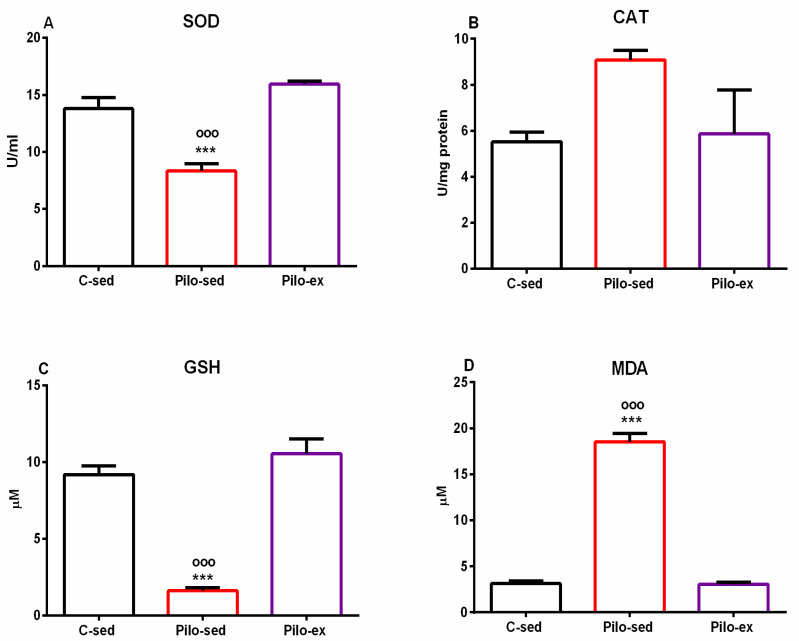
Effect of endurance training, applied before and after SE, on markers of oxidative stress in the hippocampus, SOD activity (**A**), CAT activity (**B**), GSH level (**C**), and MDA level (**D**). *** *p* < 0.001 and ^ooo^ *p* < 0.001 in comparison with the C-sed group and the Pilo-sed group, respectively.

Analysis of variance demonstrated a significant main effect of exercise [F(2, 19) = 23.874, *p* < 0.001] on superoxide dismutase (SOD) activity in the hippocampus. No differences were detected between the Pilo-ex group and the C-sed group on SOD activity, but the Pilo-sed animals had lower enzyme activity compared to the Pilo-ex group and the C-sed rats (*p* < 0.001, respectively) (Figure 8A).

Only a tendency for a main effect of exercise [F(2, 19) = 3.358, *p* = 0.059] was observed on the enzyme activity of catalase (CAT) in the hippocampus (Figure 8B).

On the glutathione (GSH) level in the hippocampus, a significant main effect of exercise was detected [F(2, 19) = 51.354, *p* < 0.001]. The post hoc test demonstrated that the Pilo-sed animals significantly lowered the GSH levels compared to the C-sed and the Pilo-ex-groups (*p* < 0.001, respectively) (Figure 8C).

ANOVA demonstrated a significant main effect of exercise [F(2, 19) = 263.588, *p* < 0.001] on malondialdehyde (MDA). The Pilo-sed animals significantly increased the levels of MDA in comparison with the C-sed and the Pilo-ex groups (*p* < 0.001, respectively) (Figure 8D).

### 2.5. Endurance Training, Applied before and after SE, Reduced the Pro-Inflammatory Cytokines Interleukin (IL)-1β Levels and Tumor Necrosis Factor (TNF)-α in the Hippocampus in the Pilo-Induced Post-SE Rat Model of TLE

**Figure 9:**
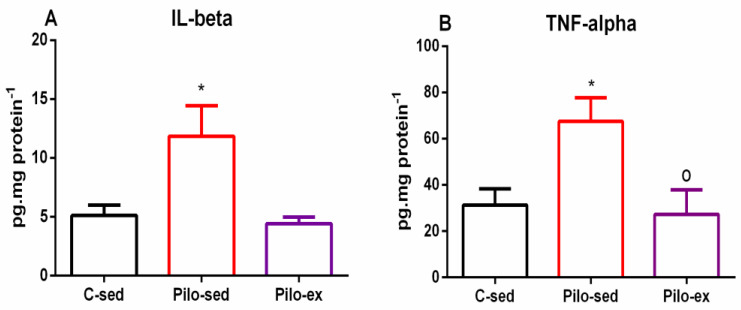
Effect of endurance training, applied before and after SE, on (**A**) pro-inflammatory cytokines IL-1β levels, * *p* = 0.024 in comparison with the C-sed group, and (**B**) TNF-α, * *p* = 0.024 in comparison with the C-sed group and ^o^ *p* = 0.0334 in comparison with the Pilo-sed group, respectively, in the hippocampus.

A significant Pilo treatment-dependent elevation of IL-1β levels in the hippocampus was detected (one-way ANOVA: [F(2, 19) = 5.432, *p* = 0.0168) in the Pilo-sed group. There was a significantly higher expression of this pro-inflammatory cytokine compared to the C-sed group (*p* = 0.024) (Figure 9A). The Pilo-ex group had similar levels of IL-1β to the C-sed group in the hippocampus, but showed a tendency for lower levels of this inflammatory signaling molecule compared to the Pilo-sed group (*p* = 0.064).

In addition, ANOVA showed a main exercise effect on the TNF-α level in the hippocampus (F(2, 19) = 5.131, *p* = 0.0159) (Figure 9B). The Pilo-sed group had elevated TNF-α levels in the hippocampus in comparison with the C-sed group (*p* = 0.024) and Pilo-ex animals (*p* = 0.0334), respectively (Figure 9B).

## 3. Discussion

It is well known that epilepsy leads to cognitive and behavioral disturbances, which can vary in every patient [9]. Experimental research with different models of epilepsy plays a key role in the exploration of the pathogenesis of epilepsy, its comorbidities and possible mechanisms underlying them, as well as finding tools to overcome them. In the current study, a pilocarpine-induced post-SE rat model of TLE provoked sustained seizures that led to impaired cognitive abilities and mood alterations. We found that endurance training, applied before Pilo treatment, suppressed the convulsant-induced SE onset in more than half of the rats, while in the rest of them, this treatment mitigated the progression to SE. Our results agree with previous reports that this alternative approach exerts a protective effect and can increase the latency and shorten the duration of Pilo-induced SE [23].

Further, we found that continuation of exercise protocol after SE had a beneficial effect on the development of spontaneous motor seizures and their consequences on cognitive abilities. The reduced seizure activity during the chronic state was also observed in a protocol with long-term aerobic exercise applied after Pilo-induced SE in the rat [22]. In the present study, we report that the applied endurance training protocol, used before and after SE, exerted a pronounced beneficial effect on several cognitive domains by neutralizing the negative effects of epilepsy. Aerobic long-term treadmill running facilitated the processes of learning and formation of both short- and long-term memories in the step-down test by increasing the time spent on the platform. Hippocampal-dependent spatial memory, a cognitive task based on gaining rewards, was also improved during the chronic phase of TLE. This effect was observed by decreasing the time for fulfillment of the task and the number of errors in the RAM. Finally, endurance training during epileptogenesis improved the associative memory in the ORT. Experimental and clinical data have shown that physical exercise improves cognitive functions and motor performance in a healthy brain at all ages [9,24]. Our data are in agreement with recently published evidence, which showed its protective effects on cognitive performance in different models of epilepsy. It could be applied as an alternative non-pharmacological approach in the treatment of epilepsy [25,26,27].

The emotional disturbance associated with increased anxiety and depression in patients with epilepsy is more often observed than in the general population [28]. In our experiment, we found that the aerobic exercise, applied before and during epileptogenesis, managed to relieve anhedonia, calculated by the preference for drinking sweet solution, which indicates a depressive-like behavior. The impulsive-like behavior was characterized by decreased anxiety levels in epileptic rats, and was not alleviated by the endurable training. Data on the effects of physical exercise on these symptoms in epileptic conditions are still very scarce. Experimental and clinical studies revealed the beneficial effect of treadmill running on anxiety- and depressive-like behavior in different experimental models, including sleep deprivation, direct social defeat, and post-traumatic stress disorder [29,30,31,32]. The present results are in accordance with our previous data on spontaneous hypertensive rats (SHR) with epilepsy [33]. We reported that the endurance training, applied before the onset of SE in SHR, reduced the frequency of SMS but was ineffective in correcting impulsive-like behavior. The beneficial effect of aerobic exercise on affective responses was reported earlier by Epps et al. [34] in SwLo rats, a strain which is vulnerable to limbic seizures and comorbid depressive-like behavior. Some possible mechanisms involved in these effects of aerobic training include increased levels of beta-endorphins, leading to a “feel-good” effect, and increased release of catecholamine, beneficial for the adaptive processes towards stress [35,36]. Experimental studies in agreement with our previous research have demonstrated that increased hippocampal levels of serotonin are another favorable mechanism of both treadmill and voluntary wheel running on depressive-like behavior [33,37]. Moreover, brain serotoninergic deficiency is supposed to be a common pathogenic mechanism underlying epilepsy and depression [38]. A recent study has demonstrated that gliosis and microglia activation might have a key role in the pathophysiology of epilepsy-associated depression [39].

The brain is extremely vulnerable to the production of free radicals, due to oxidative stress and to the high content of polyunsaturated acids and iron load, as well as the presence of many aerobic metabolic processes. Studies revealed that oxidative stress was essential in numerous neurological diseases, especially epilepsy [40]. In our experiment, we found that seizure activity increased oxidative stress, demonstrated by diminished SOD activity, reduced GSH levels, and elevated MDA levels in the hippocampus, which is in agreement with other studies that have found reduced antioxidant activity [40,41,42]. Our data showed that moderate long-term aerobic training during epileptogenesis ameliorated lipid peroxidation and restored the activity of SOD and the levels of GSH. The previous report supports our finding that the CAT activity in the hippocampus increased after Pilo-induced SE as cellular protection versus the increased production of ROS, which complies with our results [42]. We found that physical exercise managed to almost restore the CAT activity near control levels. A few recent studies have demonstrated that aerobic training can decrease oxidative stress and is effective in the treatment of impaired brain function due to free radical production [43]. Some reports revealed the neuroprotective and antioxidant effects of physical exercise before the induction of SE with kainic acid [21]. Moreover, it was found that the activated antioxidant system resulting from exercise effectively reduced epileptiform activity [43]. Additionally, free radical production induced by epileptic seizures and subsequent oxidative damage to cellular lipids, proteins, and DNA has been pointed out as a significant contributor to the observed cognitive impairment and mood changes [44,45,46,47].

Microglial and astrocytic activation plays a crucial role in the neuroinflammation of the central nervous system [48]. An increased expression of various pro-inflammatory cytokines, such as IL-1β, IL-2, IL-6, IL-8, IL-18, and TNF-α, has already been associated with the process of epileptogenesis. The SE can activate chemokines, prostaglandins, and some enzymes, such as cyclooxygenase-2 and nitric oxide synthase. All of these alterations lead to structural and functional changes in the brain, which subsequently increase neuronal excitability and seizure susceptibility, decrease the seizure threshold, and can lead to the breakdown of the blood–brain barrier [49,50]. In agreement with other studies, we found that epileptogenesis increased levels of some major pro-inflammatory cytokines, such as TNF-α and IL-1β, in the rat hippocampus. Previous reports have demonstrated that microglia were extensively activated in the brain tissues of epilepsy-induced animal models and patients with drug-refractory epilepsy, while ameliorating microglia activation and decreasing the excitotoxin-induced brain damage [49,51]. The effect of aerobic training on inflammation, especially in epileptic conditions, is still under exploration nowadays, and the data are still very insufficient. We found that aerobic exercise had a pronounced anti-inflammatory effect, and decreased the elevated levels of both cytokines in the hippocampus. Several studies observed that physical training downregulated the expression of some pro-inflammatory cytokines (IL-1β, IL-18, TNF-α) and improved cognitive performance in different models of Alzheimer’s and Parkinson’s diseases, as well as patients [52,53,54,55,56]. For that reason, inflammation has been considered an important key mechanism related to the pathogenesis not only of epilepsy, but of its comorbidities. That is why preventing microglial activation or decreasing already elevated markers of inflammation is likely to alleviate cognitive disturbances and mood changes in epileptic patients.

## 4. Materials and MethodsV 

### 4.1. Reagents

Pilocarpine (Sigma Aldrich, Hamburg, Germany); diazepam (Sopharma, Sofia, Bulgaria); scopolamine methyl nitrate (Sigma Aldrich, Hamburg, Germany); phenazine methosulfate (PMS), nitroblue tetrazolium (NBT), trichloroacetic acid (TCA), thiobarbituric acid (TBA), 5,5′-dithio-bis-(2-nitrobenzoic acid) (DTNB); TNF-α Rat ELISA Kit-Invitrogen-Thermo Fisher Scientific, Vienna, Austria, and IL-1β Rat ELISA Kit-Invitrogen-Thermo Fisher Scientific, Vienna, Austria. 

### 4.2. Animals

Fifty-two adult Wistar rats used in the study were housed under standard environ-mental conditions (12:12-h light/dark cycle, temperature 23 ± 1 °C). Tap water and laboratory chow were delivered ad libitum. The experiments were conducted following the Guidelines of the European Commission on Protection and Humane treatment of Laboratory Animals (Directive 86/609/EEC). Permission for the study was obtained from the Bulgarian Food Safety Agency № 206/01.10.2018 and from the Ethical Committee on Human and Animal Experimentation of Medical University–Plovdiv 1/28.02.2019.

### 4.3. Study Timeline, Aerobic Training Protocol, and Induction of SE

#### 4.3.1. Aerobic Training Protocol

Rats were randomly divided into two groups: sedentary (sed) and exercised (ex). Exercised animals were trained on a treadmill (EXER-3R Treadmill, Columbus Instruments, Columbus, OH, USA) five days a week with a speed of 16 m/min, at a track elevation of 5° (about 55–60% of VO_2_max), for 4 weeks [57,58]. The exercise intensity was below the lactate steady state during the treadmill running, indicating that this type of training is aerobic [59]. The running time, for the exercised rats, was 20 minutes on the 1st day, and it increased slowly by 5 minutes each following day until it reached 40 min. Sedentary rats were only placed on the treadmill for five minutes, five days a week, without running, so that they could experience stress stimuli similar to those of the training rats [60].

After one month of running, SE was induced in all exercised rats and half of the sedentary animals. After that, rats were divided into three groups (n = 16 each) as follows: (1) C-sed (controls); (2) Pilo-sed group (sedentary rats injected with Pilo 320 mg/kg i.p.); and (3) Pilo-ex group (exercised rats and injected with Pilo 320 mg/kg i.p.). Ten days after SE, the animals were given special care in order to increase their survival.

**Figure 10:**
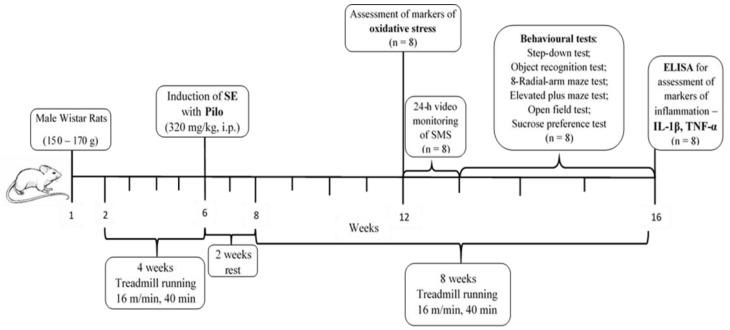
Timeline of the experimental protocol.

After this period of rest, animals started to run again on the treadmill with a velocity of 8 m/min for 10 minutes at the beginning, and gradually, in the next ten days, the intensity and time were increased until they reached 16 m/min for 40 min daily. After four weeks of exercise on the treadmill, half of the rats from each group (n = 8) were decapitated and brains were removed for assessment of oxidative stress markers. From the fifth week on, the rats were subjected to a battery of tests. The rats were trained on the treadmill in the morning, and with a 3 h interval of rest, they were given a test for behavioral assessment. At the end of the 8th week, rats were decapitated. The experimental design is demonstrated on Figure 10.

#### 4.3.2. Induction of SE with Pilocarpine

As previously described, SE was induced by an intraperitoneal injection of Pilo (320 mg/kg), 30 minutes after administration of scopolamine methyl nitrate 1 mg/kg i.p [61,62]. Seizure severity was evaluated based on the Racine scale [63].

### 4.4. Video Monitoring of Spontaneous Motor Seizures (SMS)

The spontaneous motor seizures were registered (24 h/daily) with video recording (infrared-sensitive camera, S-2016, AVTECH, Taiwan, no. AVC307R) and a computer system. The epileptic rats were placed in individual and labeled Plexiglas cages eight weeks after SE. The data from the detected seizures at stage 4 from video recordings were analyzed offline.

### 4.5. Behavioural Tests

#### 4.5.1. Step-Down Test

The step-down test for passive avoidance with negative reinforcement was performed in order to evaluate the passive learning and memory abilities of rats, as described previously [64]. In brief, the rats were placed on a vibrating platform, and the reaction latency was measured when they tried to climb down from the platform.

#### 4.5.2. Object Recognition Test

The object recognition protocol (ORT) consisted of a sample session and a test session. The ORT was carried out as previously described [62,65]. The time (s) needed to explore the familiar (A) and novel (B) objects was recorded during the test session. The discrimination index (DI) was calculated by determining the difference in exploration time between the novel and familiar objects, and dividing it by the total object exploration time: (TB − TA)/(TB + TA).

#### 4.5.3. Sucrose Preference Test

A sucrose preference test was performed in order to assess depressive-like behavior, as previously described [62,65]. The test was carried out on 3 consecutive days, and the consumed sucrose solution and water (in mL) were measured on the 3rd (test) day. The sucrose preference (%) was calculated for each rat as follows: [sucrose consumption (mL)/(sucrose consumption (mL) + water consumption (mL)) × 100%].

#### 4.5.4. Eight-Radial Arm Maze Test

The spatial memory was assessed with an eight-radial arm maze (RAM) apparatus (Harvard Biosci. Comp., Holliston, MA, USA), as described recently by our team [66,67]. The number of working memory errors (WMEs) was recorded (repeated entry into an empty arm where the pellet had already been retrieved). The time to eat the eight pellets was also estimated or the test was interrupted in 10 min.

#### 4.5.5. Open Field Test

The motor activity and anxiety behavior were explored in the open field (OF) apparatus (100 × 100 cm × 60 cm) made by polystyrene. The test was carried out for 5 minutes, according to the protocol described in our previous work [68]. The distance traveled in the periphery (cm), distance, and time (s) in the central zone were measured through the automatic video tracking system (SMART PanLab software, Harvard Apparatus, Holliston, MA, USA).

#### 4.5.6. Elevated Plus Maze Test

As previously reported, the elevated plus maze test was performed in order to evaluate the anxiety-like behavior [62,65]. In brief, the following behavioral parameters were determined: duration and number of entries in open arms and closed arms. The anxiety index (AI) was calculated as follows: AI = 1 − [(open arms time/total time) + (number of entries in open arms/total number of entries)/2]. The alleys of the maze were cleaned with ethanol solution (60% volume) after the removal of each rat [63,65].

### 4.6. Markers of Oxidative Stress in the Hippocampus

#### 4.6.1. Preparation of Tissue Homogenate

The hippocampus was homogenized in an ice bath with phosphate buffer (pH = 7), and a tissue: buffer ratio of 1:25. The homogenate was then centrifuged at 10,000× *g* for 5 min at 4 °C. The obtained supernatant was transferred into another test tube and used for the analysis of GSH and MDA concentration, as well as for determining the activity of SOD and CAT.

#### 4.6.2. Protein Content

Protein content was determined as previously described by our team [61,62].

#### 4.6.3. MDA Assay

The reaction mixture, composed of 290 µL 0.1M phosphate buffer (pH = 7.4), 100 µL supernatant, 100 µL ascorbic acid (100 µM), and 10 µL iron trichloride (100 µM), was incubated for 1 h at 37 °C. At the end of the incubation time, 500 µL of each 10% TCA and 0.67% TBA were added. Test tubes were placed in a boiling water bath for 20 minutes, taken out, placed on ice, and centrifuged at 2500× *g* for 10 min. The absorbance of the supernatant was measured against a blank sample at 535 nm.

#### 4.6.4. SOD Activity Assay

The activity of SOD was measured based on the Kakkar method [69]. The reaction mixture was composed of 600 µL 0.052 mM phosphate buffer (pH = 8.16), 50 µL PMS (186 µM), 150 µL, NBT (300 µM), and 150 µL supernatant. The reaction was started by the addition of 100 µL NADH (780 µM), and stopped after one minute with 500 µL glacial acetic acid. Absorbance was measured against a blank sample, at 560 nm. SOD activity was calculated as U/mg protein.

#### 4.6.5. CAT Activity Assay

The activity of CAT was measured using 0.036% solution of hydrogen peroxide in 50 mM phosphate buffer (pH = 7). Then, 100 µL of the supernatant was added to 2.9 mL of the hydrogen peroxide solution, and the changes in absorbance were monitored for three minutes at 240 nm. CAT activity was calculated as U/mg protein.

#### 4.6.6. GSH Assay

The GSH was measured according to Ellman’s assay. To 50 µL of the homogenate, we added 50 µL of 10% TCA. The mixture was centrifuged at 1500× *g* for 15 min and the supernatant was taken for analysis. To 200 µL, we added 262 mM Tris-HCl (pH = 8.9), 20 µL DTNB, and 50 µL of the supernatant. Absorbance was measured after 15 minutes of reaction time, against a blank sample at 412 nm. 

### 4.7. Measurement of IL-1β and TNF-α in the Hippocampus

The Bradford method was used for the evaluation of protein content [70]. The measurement of the IL-1 beta and TNF-alpha was executed with invitrogen ELISA kits, according to the manufacturer’s instructions. The concentration was expressed as pg/mg protein^−1^.

### 4.8. Statistical Analysis

Experimental results were presented as mean ± S.E.M. After testing for assumptions of normality of data distribution and homogeneity of variance, one-way ANOVA with factor treatment was used for the step-down test, ORT, OF, EPM, SPT, oxidative stress, and inflammatory markers. Data on RAM were analyzed by two-way ANOVA. Post hoc comparisons via the Bonferroni test in case of justification were used. Statistically significant differences were accepted at *p* ≤ 0.05. Student’s *t*-test was applied for the analysis of SMS data. The analysis was conducted using the SigmaStat^®^ (version 11.0.) statistical package.

## 5. Conclusions

The endurance training, applied before and after SE, mitigated the Pilo-induced SE and the development of epileptogenesis. The beneficial effect of this alternative treatment resulted in lower SMS, improved memory responses, and reduced anhedonia. In addition, endurance training mitigated the oxidative stress and inflammation in the hippocampus of the epileptic rat. Our findings suggest that the exercise approach predisposes subjects to crucial plastic changes that can lead to SE resistance and further development of epileptogenesis. Future studies are needed to elucidate the underlying mechanisms and signaling molecules involved in exercise-induced neuroprotection in epilepsy.

## Data Availability

Data available in a publicly accessible repository that does not issue DOIs.

## Abbreviations

ANOVA	analysis of variance
CAT	catalase
DTNB	5,5′-dithio-bis-(2-nitrobenzoic acid
EPM	elevated plus maze
Ex	exercised
GSH	reduced glutathione
IL-1β	interleukin-1β
IL-6	interleukin-6
MDA	malondialdehyde
NBT	nitroblue tetrazolium
OP	open field
ORT	object recognition test
Pilo	pilocarpine
PMS	phenazine methosulfate
RAM	eight-radial arm maze
SE	status epilepticus
Sed	sedentary
SOD	superoxide dismutase
SMS	spontaneous motor seizures
TBA	thiobarbituric acid
TCA	trichloroacetic acid
TNF-α	tumor necrosis factor-α
WME	working memory errors
